# Supplementary material for: Alterations in Calcium Handling Are a Common Feature in an Arrhythmogenic Cardiomyopathy Cell Model Triggered by Desmosome Genes Loss
Source: Int J Mol Sci. 2023 Jan 20;24(3):2109. doi: 10.3390/ijms24032109 (PMC9917020; doi:10.3390/ijms24032109)
Supplement: Supplementary file 1 [file ijms-24-02109-s001.zip › ijms-2132379-supplementary.pdf]

Supplemental material

TABLES

Table S1. Primers, PCR conditions and kits for Sanger sequencing.

	**Primer sequence**	**PCR program**	**PCR Kit**
PKP2_FW	CAGTGTAGATTGGCACGTTTGT	PCR60	Gotaq (M743A, Promega)
PKP2_RV	GGGAAAGGAGACCACTTGAGA		
DSP_FW	TGAGCGGCTCTCTTG	Touchdown	Hotstart buff A + enhancer + DMSO (KK5515, KAPA biosystems)
DSP_RV	CCTGCGCTTCCGTGACC		
DSG2_FW	GGACCTGCCCAGGAGGAT	Touchdown	Hotstart buff GC (KK5515, KAPA biosystems)
DSG2_RV	TGCCTACCCTGCTCTCCC		
DSC2_FW	AGTGGCCGATTCGAGTCTTT	PCR60	Gotaq (M743A, Promega)
DSC2_RV	TCGCGCGCCTATTACAGCT		

Table S2. CRISPR/Cas9 edited clones of the three desmosomal genes studied.

NI: Not identified (homozygous mutation or big deletion).

Desmosomal gene	Clones	Allele 1 genotype	Allele 2 genotype
PKP2	27	c.325ins1	c.324del2
	31	c.326ins1	c.322del2
	32	c.325ins1	c.324del1
	35	c.325ins1	c.325del4
DSG2	1	c.226del4	c.229ins1
	6	c.220del 30+ins7	NI
	11	c.220del7	c.228del1
	14	c.221del10	c.229ins1
DSC2	14	WT	c.313del14
	16	WT	c.326ins1
	18	c.324del4	NI
	21	c.325del1	NI

Table S3. RQ values from qPCR of desmosomal, calcium handling and electrical genes in PKP2, DSG2 and DSC2 edited clones. ND: No Data.

PKP2-KO	PKP2	DSP	DSC2	DSG2	JUP	SCN5A	CX43	ATPA2A	CASQ2	RYR2	SLC8A1	TRDN	ANK2	CACNA1C	PLN
**WT9**	0,80	0,79	0,75	0,61	0,76	0,67	0,88	0,91	0,73	1,08	0,95	1,35	1,54	1,00	1,000
**WT88**	0,84	1,77	0,96	1,82	0,31	2,41	1,76	1,67	1,66	1,57	1,49	1,12	1,30	1,15	ND
**WT97**	0,78	0,67	0,39	0,52	1,27	0,37	0,57	0,53	0,76	0,63	0,57	0,85	1,65	1,05	0,927
**WT140**	1,00	1,00	1,00	1,00	1,00	1,00	1,00	1,00	1,00	1,00	1,00	1,00	1,00	0,88	0,931
**PKP2-27**	0,13	0,34	0,00	0,60	0,59	0,30	0,21	0,22	0,11	0,36	0,21	0,16	0,02	0,33	1,188
**PKP2-31**	0,19	0,52	0,00	0,25	0,56	0,13	0,13	0,13	0,12	0,23	0,18	0,40	0,01	0,45	1,298
**PKP2-32**	0,16	0,53	0,00	0,68	0,77	0,32	0,23	0,31	0,23	0,39	0,25	0,24	0,02	0,26	1,745
**PKP2-35**	0,09	0,31	0,00	0,47	0,37	0,16	0,15	0,15	0,13	0,23	0,15	0,23	0,02	0,27	1,171

DSG2-KO	PKP2	DSP	DSC2	DSG2	JUP	SCN5A	CX43	ATPA2A	CASQ2	RYR2	SLC8A1	TRDN	ANK2	CACNA1C	PLN
**WT9**	1,00	1,00	1,00	1,00	1,00	1,00	1,00	1,00	1,00	1,00	1,00	1,00	1,00	1,00	1,000
**WT88**	0,77	0,83	0,42	0,96	0,85	1,44	0,55	0,53	1,13	0,62	0,92	0,93	0,79	1,15	ND
**WT97**	1,43	1,36	0,65	0,81	0,66	1,62	0,46	0,60	1,71	0,56	0,99	1,36	2,53	1,05	0,927
**WT140**	0,89	0,77	0,72	1,41	0,98	2,13	0,79	0,89	1,20	0,64	1,38	1,21	1,13	0,88	0,931
**DSG2-1**	1,19	0,86	0,00	0,91	0,53	0,55	0,09	0,21	0,07	0,56	0,40	0,17	0,02	0,27	1,237
**DSG2-6**	1,75	0,90	0,00	0,33	0,56	0,39	0,14	0,20	0,62	0,49	0,25	1,20	0,04	0,49	1,614
**DSG2-11**	2,67	1,78	0,00	0,42	0,61	0,98	0,24	0,24	0,54	0,76	0,91	0,62	0,03	0,34	1,506
**DSG2-14**	1,66	1,73	0,00	1,86	0,61	1,16	0,22	0,37	0,24	0,97	0,75	0,40	0,06	0,20	0,732

DSC2-KO	PKP2	DSP	DSC2	DSG2	JUP	SCN5A	CX43	ATPA2A	CASQ2	RYR2	SLC8A1	TRDN	ANK2	CACNA1C	PLN
**WT9**	1,00	1,00	1,00	1,00	1,00	1,00	1,00	1,00	1,00	1,00	1,00	1,00	1,00	1,00	1,000
**WT82**	1,10	2,51	1,39	3,27	2,44	4,32	2,52	2,09	2,15	1,51	2,02	1,93	0,85	1,15	ND
**WT97**	1,04	0,87	0,55	0,83	1,51	0,56	0,70	0,62	0,98	0,61	1,41	1,17	0,91	1,05	0,927
**WT140**	1,20	1,33	1,30	1,61	1,31	1,64	1,48	1,20	1,43	1,09	0,43	0,65	0,69	0,88	0,931
**DSC2-14**	0,84	0,69	0,00	1,43	0,85	0,59	0,47	0,50	0,35	0,51	0,11	0,07	0,01	0,36	0,893
**DSC2-16**	0,71	0,53	0,00	0,35	0,40	0,20	0,14	0,20	0,31	0,22	0,31	0,17	0,01	0,34	1,478
**DSC2-18**	1,00	1,31	0,00	2,13	1,26	0,59	0,42	0,56	0,37	0,79	0,44	0,14	0,01	0,47	1,000
**DSC2-21**	1,10	0,49	0,00	1,44	1,20	0,41	0,38	0,24	0,24	0,27	0,27	0,64	0,00	0,63	1,431

Table S4. p-value from qPCR and WB results from desmosomal expression genes in PKP2, DSG2 and DSC2 edited clones.

Desmosomes genes are coloured in green, calcium handling in blue and connexome in pink. The first column of each clones group corresponds to the RNA levels and the second column to protein levels. Genes that present a significant downregulation are in yellow (p-value<0,5). Those proteins that couldn't be studied due to a limitation of antibodies are in grey. UD indicates undetectable levels of protein expression in KO but that are present in WTs. *p-value of the DSG2 canonical isoform (≈135 KDa).

	PKP2-KO		DSG2-KO		DSC2-KO	
	RNA	Protein	RNA	Protein	RNA	Protein
PKP2	0,02	UD	0,05	0,02	0,34	1
DSP	0,02	0,02	0,34	0,2	0,11	0,02
DSG2	0,2	UD*	0,48	UD*	0,88	0,88*
DSC2	0,02	UD	0,02	UD	0,02	UD
JUP/PG	0,48	0,02	0,02	0,02	0,11	0,11
RYR2	0,02	0,02	0,88	0,02	0,05	0,02
CASQ2	0,02		0,02		0,02	
SLC8A1	0,02		0,02		0,05	
ATPA2A	0,02		0,02		0,02	
ANK2	0,02	UD	0,02	UD	0,02	UD
TRDN	0,02		0,11		0,02	
CACNA1C	0,02		0,04		0,61	
PLN	0,34		0,34		0,34	
CX43	0,02	0,2	0,02	0,02	0,02	0,11
NAV1.5	0,02	UD	0,05	UD	0,11	UD

Table S5. Number of obtained traces per cell line in 10mM caffeine peak.

	Clone	Number of traces
**WT**	9	230
	88	392
	97	256
	140	308
**PKP2**	27	573
	31	517
	32	446
	35	364
**DSG2**	1	324
	6	236
	11	483
	14	299
**DSC2**	14	316
	16	524
	18	430
	21	234
**DSP**	N-truncated	260
	KO	385

Table S6. p-value from Hierarchical analysis of 10mM caffeine peak

*significant downregulation (p-value<0,5).

	PKP2-KO	DSG2-KO	DSC2-KO
**Half Width**	0,006*	0,001*	0,00*
**Rise Time 10-50%**	1	0,30	0,36
**Decay Time 50-10%**	0,76	0,11	0,25
**Rise Time 10-90%**	1	0,01*	0,02*
**Decay Time 90-10%**	0,90	0,04*	0,08
**Decay Time 90-50%**	1	0,03*	0,02*

Table S7. Values from 10mM caffeine peak in PKP2, DSG2 and DSC2 edited clones. Values unit is ms (time).

**Mean**	Half Width	Rise time 10-50%	Decay time 50-10%	Rise Time 10-90%	Decay Time 90-10%	Decay Time 90-50%
**WT**	912,48	42,36	371,87	142,27	764,31	392,45
**PKP2**	1293,45	56,04	542,98	212,98	983,98	440,97

**DSG2**	1345,88	64,55	648,05	342,09	1202,80	554,53
**DSC2**	1384,31	63,32	607,31	326,32	1168,55	563,42
**SE**						
**WT**	65,56	7,44	75,54	37,96	103,05	36,67
**PKP2**	67,32	7,41	75,07	37,82	102,52	36,46
**DSG2**	67,50	7,43	75,43	37,92	102,92	36,61
**DSC2**	67,45	7,43	75,33	37,90	102,82	36,57

Table S8. p-value from TGFB1 in PKP2, DSG2 and DSC2 edited clones. *Significant downregulation (p-value<0,5).

	PKP2-KO	DSG2-KO	DSC2-KO
TGFB1	0,26	0,003*	0,01*

Table S9. RQ values from TGFB1 in PKP2, DSG2 and DSC2 edited clones.

	TGFB1
**WT9**	1
**WT88**	1,388
**WT97**	0,791
**WT140**	1,361
**PKP2-27**	1,811
**PKP2-31**	1,39
**PKP2-32**	1,718
**PKP2-35**	1,255
**DSG2-1**	2,111
**DSG2-6**	2,622
**DSG2-11**	3,257
**DSG2-14**	1,728
**DSC2-14**	1,63
**DSC2-16**	1,911
**DSC2-18**	2,102
**DSC2-21**	2,974

Table S10. CTs from qPCR of PPARγ expression gene in PKP2, DSG2 and DSC2 edited clones.

	CTs PPARγ	CTs Mean	CTs Standard Error
**WT9**	35,30	36,18	0,98
**WT88**	37,47		
**WT97**	36,42		
**WT140**	35,54		
**PKP2-27**	32,91	32,78	0,33
**PKP2-31**	32,31		
**PKP2-32**	33,07		
**PKP2-35**	32,82		
**DSG2-1**	31,93	31,06	1,92
**DSG2-6**	32,72		
**DSG2-11**	31,27		
**DSG2-14**	28,31		
**DSC2-14**	35,26	33,65	1,16
**DSC2-16**	33,48		
**DSC2-18**	32,49		
**DSC2-21**	33,39		

Table S11. RQ values from qPCR of *CACNA1C* and *PLN* genes in DSP edited clones.

	CACNA1C	PLN
**WT97**	1,000	1,000
**DSP28**	0,565	0,773
**DSP21**	0,836	0,315

Table S12. Values from 10mM caffeine peak in DSP clones. Values unit is ms (time).

**Mean**	Half Width	Rise time 10-50%	Decay time 50-10%	Rise Time 10-90%	Decay Time 90-10%	Decay Time 90-50%
**WT97**	889,29	38,14	337,03	141,57	698,04	361,01
**DSP28**	908,66	53,77	483,80	193,34	858,93	375,13
**DSP21**	744,37	47,19	384,16	105,46	775,29	388,47
**SE**						
**WT97**	219,23	39,77	247,86	107,84	274,09	100,23
**DSP28**	388,67	41,08	390,70	139,90	571,90	243,56
**DSP21**	180,66	25,79	88,50	48,37	99,40	88,37

FIGURES

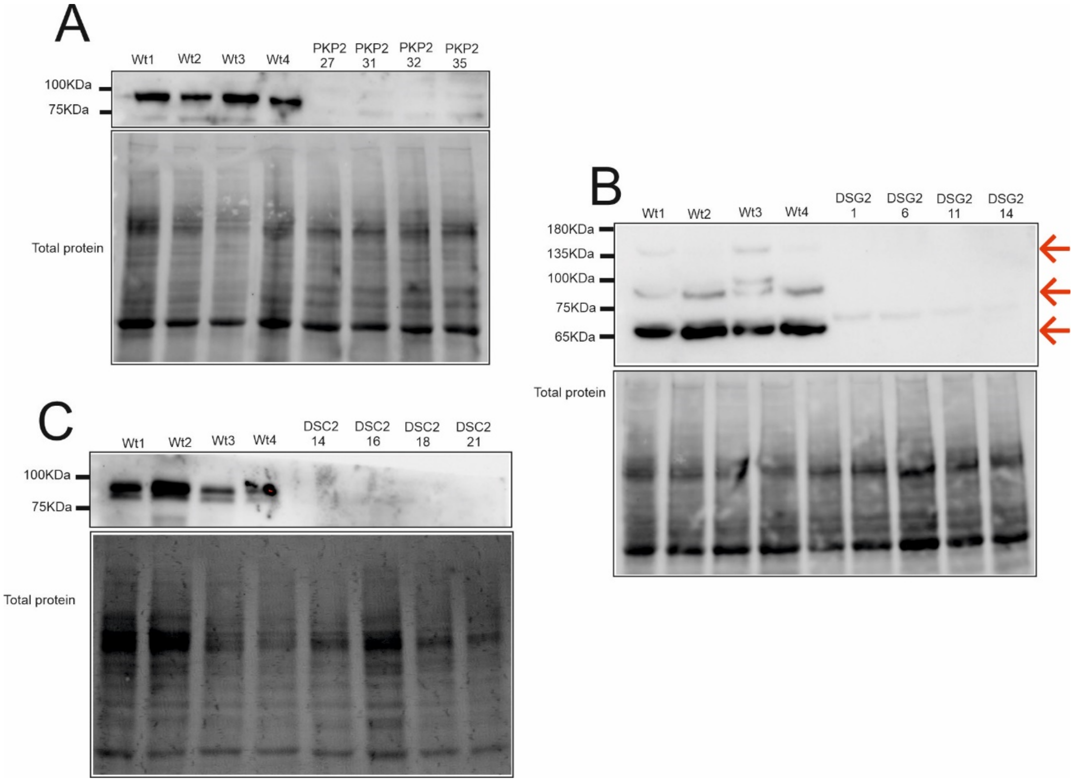

Figure S1. Protein expression levels of the edited gene in each group: PKP2 for *PKP2*-KO **(A)**, DSG2 for *DSG2*-KO **(B)** and DSC2 for *DSC2*-KO **(C)**. The DSG2 antibody recognises three isoforms (canonical 130 kDa, 80 kDa, and 65 kDa) marked with a red arrow. Published data **(Vallverdu-Prats, Int. J. Mol. Sci. 2022).**

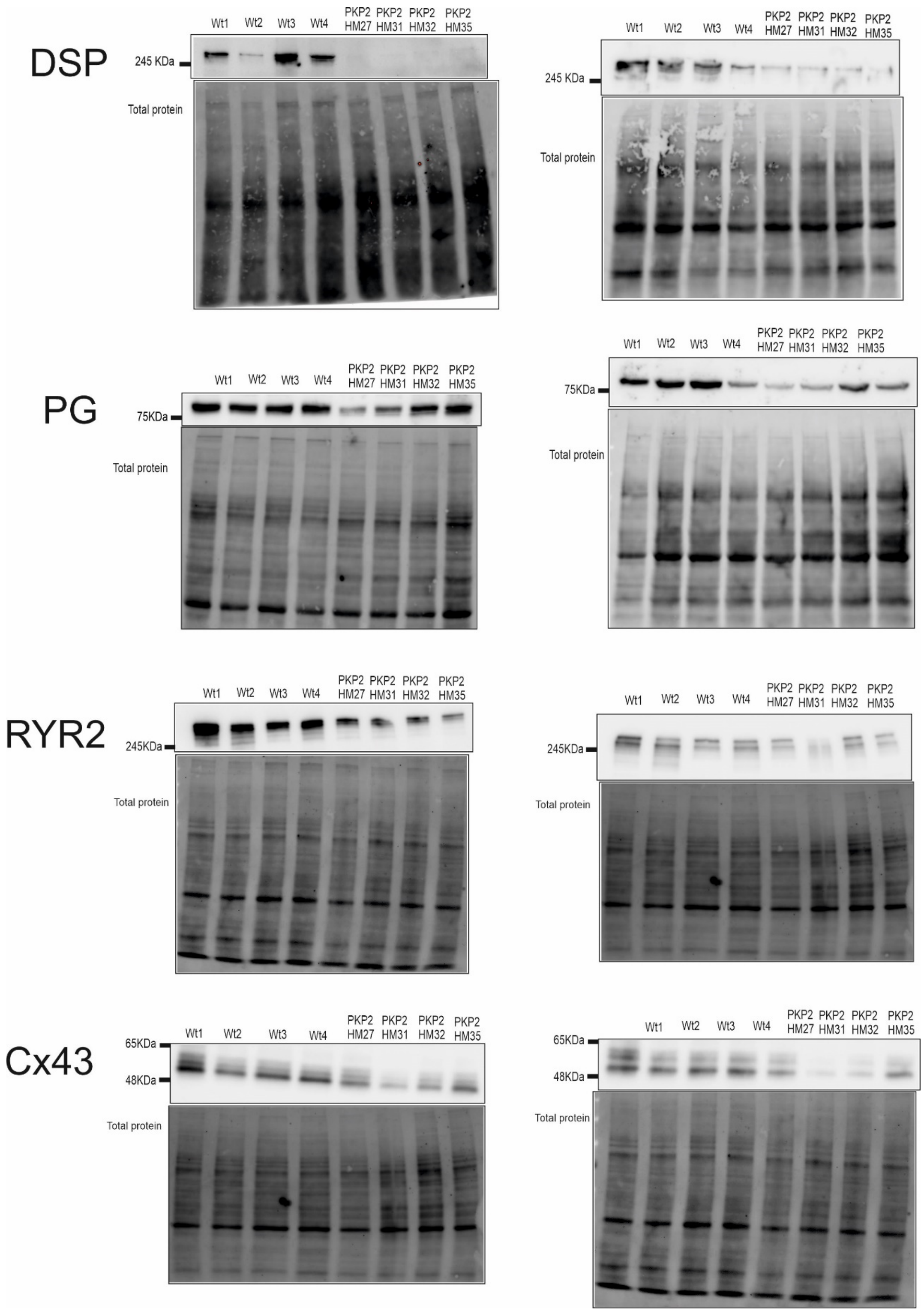

Figure S2. WB replicates of PKP2-KO clones.

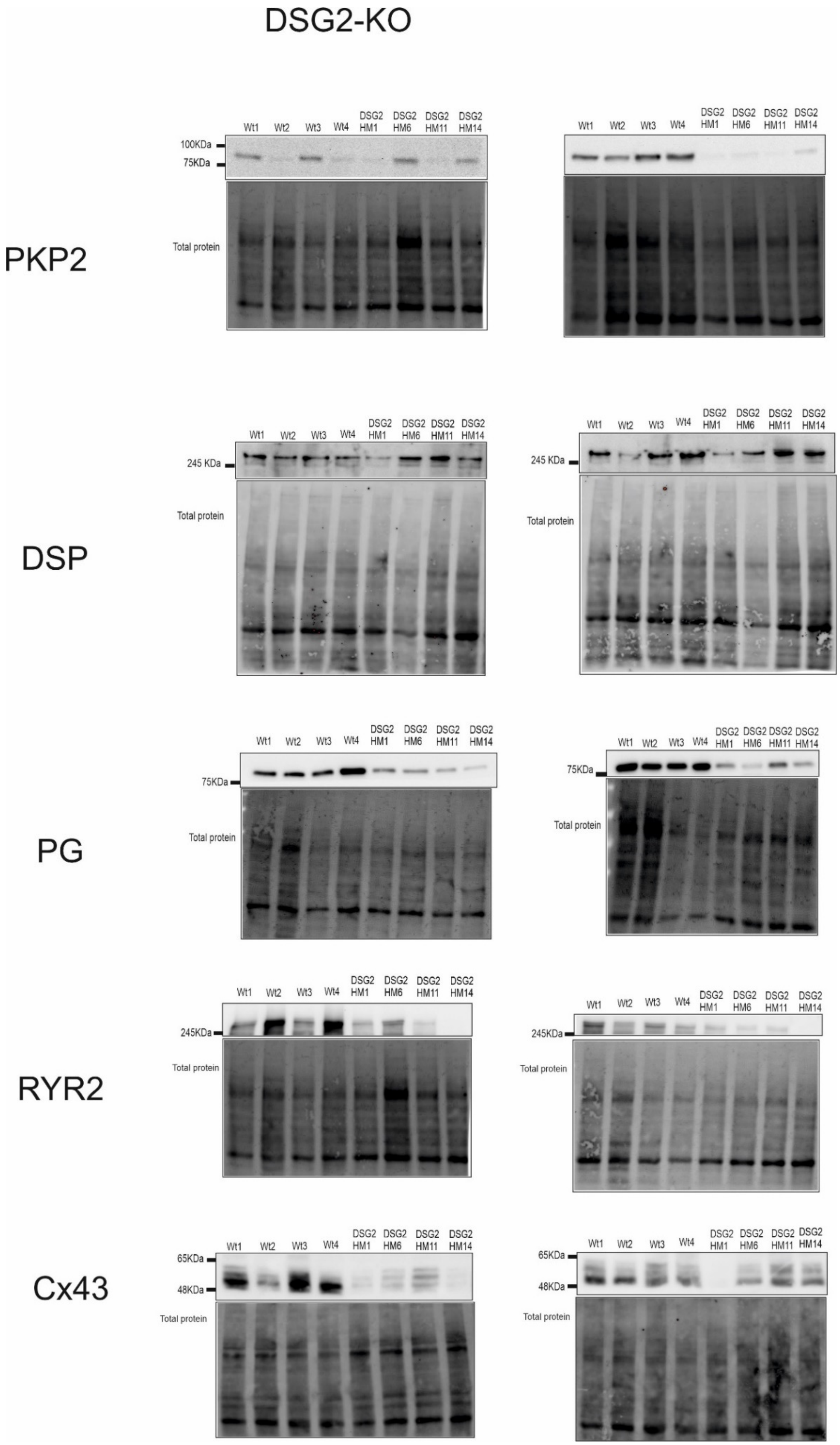

Figure S3. WB replicates of DSG2-KO clones.

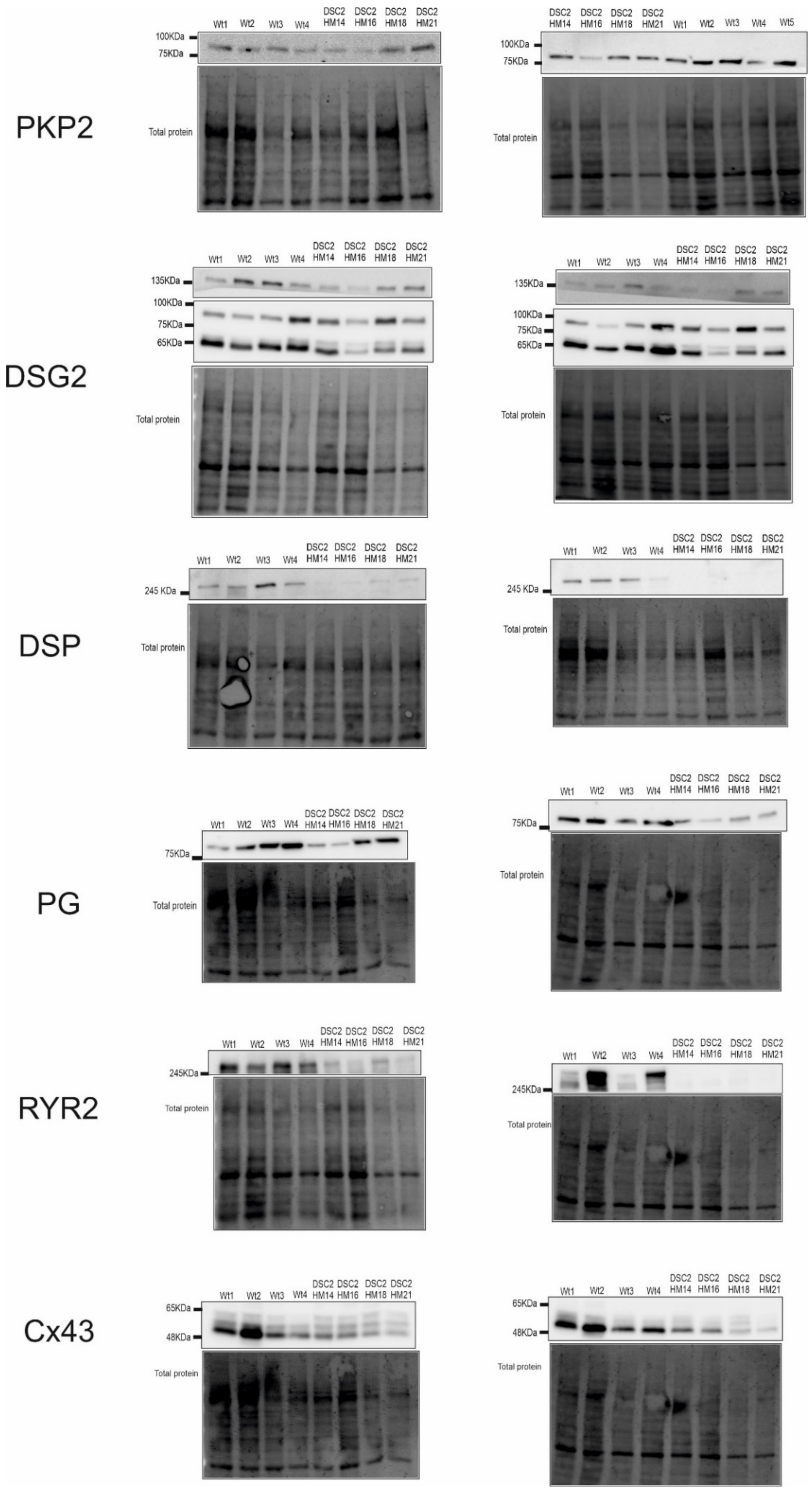

Figure S4. WB replicates of DSC2-KO clones.
